# AtXRN4 Affects the Turnover of Chosen miRNA*s in *Arabidopsis*

**DOI:** 10.3390/plants9030362

**Published:** 2020-03-13

**Authors:** Yan Liu, Wenrui Gao, Shuangyang Wu, Lu Lu, Yaqiu Chen, Junliang Guo, Shuzhen Men, Xiaoming Zhang

**Affiliations:** 1 State Key Laboratory of Integrated Management of Pest Insects and Rodents, Institute of Zoology, Chinese Academy of Sciences, Beijing 100101, China; liuyan8882@126.com (Y.L.); gaowenrui2020@126.com (W.G.); wushuangyang@ioz.ac.cn (S.W.); lulubio2018@163.com (L.L.); 15670533706@163.com (Y.C.); guojunliang2008@163.com (J.G.); 2 CAS Center for Excellence in Biotic Interactions, University of Chinese Academy of Sciences, Beijing 100049, China; 3 Department of Plant Biology and Ecology, College of Life Sciences, Nankai University and Tianjin Key Laboratory of Protein Science, Tianjin 300071, China; shuzhenmen@nankai.edu.cn; 4 Henan Normal University, Department of Life Sciences, Xinxiang, Henan 453007, China; 5 Institute of Physical Science and Information Technology, Anhui University, He fei, Anhui 230601, China

**Keywords:** small RNA, RNA turnover, XRN, Argonaute, DCP

## Abstract

Small RNA (sRNA) turnover is a key but poorly understood mechanism that determines the homeostasis of sRNAs. Animal XRN genes contribute the degradation of sRNAs, AtXRN2 and AtXRN3 also contribute the pri-miRNA processing and miRNA loop degradation in plants. However, the possible functions of the plant XRN genes in sRNA degradation are far from known. Here, we find that AtXRN4 contributes the turnover of plant sRNAs in Arabidopsis thaliana mainly by sRNA-seq, qRT-PCR and Northern blot. The mutation of AtXRN4 alters the sRNA profile and the accumulation of 21 nt sRNAs was increased. Some miRNA*s levels are significantly increased in *xrn4* mutant plants. However, the accumulation of the primary miRNAs (pri-miRNAs) and miRNA precursors (pre-miRNAs) were generally unchanged in *xrn4* mutant plants which indicates that AtXRN4 contributes the degradation of some miRNA*s. Moreover, AtXRN4 interacts with *Arabidopsis* Argonaute 2 (AtAGO2). This interaction takes place in Processing bodies (P-bodies). Taken together, our observations identified the interaction between XRN4 with AtAGO2 and suggested that plant XRN4 also contributes the turnover of sRNAs.

## 1. Introduction

Small RNAs (sRNAs) are 20–30 nucleotide (nt) noncoding RNAs that regulate the expression of target genes at the transcription or post-transcription levels [1,2]. The homeostasis of sRNAs is tightly regulated at the transcription and post-transcription levels [3,4]. Although the transcription and processing of microRNAs (miRNAs), a major sRNA for both animals and plants, have been well studied, the degradation mechanisms of miRNAs and other sRNAs remain unclear. The functions of exoribonucleases but not endoribonucleases have been proven in the turnover of sRNAs.

The turnover of sRNA is first demonstrated by its 3′ terminal modification and degradation. In plants, 3′ ends of sRNA duplexes are methylated by HUA Enhancer 1 (HEN1), which is a critical step in miRNA stabilization. The methylation of 3′ ends protects sRNAs from being degraded by 3′ truncation and uridylation [5]. HEN1 SUPPRESSOR1 (HESO1) and UTP: RNA Uridylyltransferase 1 (URT1) act as 3′ uridylation transferases synergistically in the 3′tailing [6,7]. Atrimmer 2 (ATRM2) acts in the 3′-5′ degrading of unmethylated miRNA/miRNA* duplexes. Loss-of-function mutants of ATRM2 restored levels of a subset of miRNAs which were degraded in hen1 mutants and they observed a marked increased trimming of several miRNA*s but not miRNAs in ATRM2 malfunction backgrounds which infers the existence of another yet unknown exoribonuclease [8]. SMALL RNA DEGRADING NUCLEASE (SDN) family were identified as 3′-5′ exonucleases that degrade single-strand sRNAs in vivo and in vitro. SDN mutants accumulate higher levels of miRNAs and purified GST-SDN1 protein degrades single-strand sRNA in vitro with size of 17, 18, 20, 21, 22, 23, 24 and 27nt and yield an approximately 8–9 nt end products [9]. In animals, a number of 3′-5′ exribonucleases have been reported to function in small RNA 3′ processing and degradation including MUT-7 and Poly(A)-specific Ribonuclease (PARN-1) in C. elegans, Nibbler in Drosophila [10,11]. RRP41degrades miR382 and some miRNAs in human embryonic kidney (HEK293T) cells [12]. ERI-1 degrades siRNA with 2-nt 3′ overhangs [13]. The exoribonuclease DIS3L2 specifically binds and degrades uridylated RNA substrates such as uridylated pre-let-7, 7SL, and snRNA, but its homologous in Arabidopsis is inactive in Col-0 background. 

The 5’-3’ degradation of sRNAs has been partially studied in animals. The depletion of XRN1 and XRN2, two 5’-3’ exoribonucleases encoded by *Caenorhabditis elegans*, leads increased accumulation of miRNAs and a subset of miRNA*s without the alterations of pri-miRNAs and pre-miRNAs. [14,15]. These observations indicate that CeXRN1 and CeXRN2 contribute to the degradation of sRNAs. Homo sapiens XRN1, but not HsXRN2, also contributes to the degradation of some miRNAs [12]. Recent studies showed that the accumulation of pri-miRNAs and corresponding mature miRNAs are increased and decreased in *xrn2-1* respectively, which indicates that XRN2 promotes the processing of pri-miRNAs [16]. AtXRN2, AtXRN3, and AtXRN4 are the homologue genes encoded by *Arabidopsis*. AtXRN2 and AtXRN3 redundantly contribute the processing of pre-rRNA and the maturation of rRNA [17]. AtXRN4 is the closest homolog of yeast Xrn1p [18] AtXRN4, also referred to as EIN5, contributes the transduction of ethylene signal [19]. Function as a 5′→3′ exoribonuclease, AtXRN4 is required for regulation of the F-box protein EBF1/2 which negatively regulate the key component of ethylene pathway, EIN3 [20,21]. Further study showed that ATXRN4 interacts EIN2 when it targets EBF1 3′ UTR to cytoplasmic P-bodies [22]. AtXRN2, AtXRN3, and AtXRN4 are all involved in miRNA pathway. AtXRN2 and AtXRN3 eliminate the pri-miRNAs loop remnants after DCL1-mediated cleavage, while AtXRN4 contributes the degradation of the 3′ end of a subset of miRNA-mediated cleavage fragment [17,23]. Similar to miRNA-mediated decay in plants, posttranscriptional gene silencing (PTGS) is a process that involved RNA decay, small interfering RNA (siRNAs) and XRN activity [24]. The accumulations of decapped transgenic RNA and sRNAs were increased in *ein5* mutant plants [25]. Further study uncovered that EIN5 is a repressor for transgene and endogenous PTGS [26]. Upon the dysfunction of 5′-3′(EIN5) and 3′-5′ (SKI-Exosome) RNA decay pathways, aberrant mRNAs are amplified by SGS3/RDR6 and processed into a large number of 21- to 22-nt endogenous siRNAs. These siRNAs are termed as coding transcripts derived small interfering RNA (ct-siRNA). However, the direct function of AtXRNs in the turnover of sRNAs is still unknown. 

Argonaute (AGO) is the key component of RNA silencing [27]. Associated with sRNAs, AGO regulated the expression of sRNA target genes at transcriptional or post-transcriptional levels.

*Arabidopsis* encodes ten AGOs that are classified into three subgroups based on their sequence similarity: AGO1/5/10, AGO2/3/7, and AGO4/6/8/9 [28]. Most *Arabidopsis* miRNAs contain a 5′ terminal U and are predominantly loaded into AGO1 for function. In miRNA*s usually contain a 5′ terminal A and were predominantly loaded into AGO2 [29]. AGO2 shows both additive and overlapping activity with AGO1. Both of them contribute to plant defense against *Pseudomonas* and a broad range of viruses. Upon *Pseudomonas* infection, the loading of miR393b* into AGO2 was increased. miR393b* targets MEMB12 and the decreased accumulation of MEMB12 upon bacterial infection leads the increased secretion of antimicrobial protein PR1. Interestingly, the accumulation of miR393 is also increased upon bacterial infection and it corporately increase plant immunity by regulating auxin signaling pathway [30].

To detect the function of AtXRN4 in the sRNA degradation, we constructed sRNA libraries with Col-0 WT and xrn4 mutant plants. sRNA sequencing was then conducted with these sRNA libraries. The mutation of AtXRN4 altered the sRNA profile and the accumulations of miRNA*s was significantly increased in *xrn4* mutant plants. However, the accumulations of pri-miRNAs and pre-miRNAs were not generally altered which argue against the function of AtXRN4 in miRNA processing. These results indicated that AtXRN4 contributes to the turnover of some miRNA*s. Previous studies demonstrated that plant miRNA*s majorly associate with AtAGO2 [29,30]. We then determined the interaction of AtXRN4 with AtAGO2 and uncovered the association of these two proteins. Moreover, their interaction sites co-localize with the subcellular localization sites of DCP1, a marker protein of P-bodies. Our observations thus reveal the association between AtXRN4 with AtAGO2 and indicate that AtXRN4 affects the degradation of some *Arabidopsis* miRNA*s.

## 2. Results

### 2.1. AtXRN4 Mutation Changes the sRNA Profiles

Considering the significant roles of XRN genes in animal sRNA turnover, the role of AtXRN2 and AtXRN3 in pri-miRNA processing and miRNA loop degradation, and the key role of AtXRN4 in the degradation of a subset of 3’ fragments of miRNA targets, we decided to determine the function of AtXRN in plant sRNA turnover. Our phylogenetic tree analysis of the XRN orthologue proteins proved thatAtXRN2, AtXRN3, and AtXRN4 are all orthologs of CeXRN2rather than CeXRN1 (Figure S1). Moreover, AtXRN4 contains all the conserved exoribonuclease motifs (Figure S2) and showed a mild phenotype with serrated leaves (Figure S3b), multiple fruits emanate from the same node (Figure S3c), and late flowering (Figure S3d) which have described previously [24,31] Because of the important functions of AtXRN4 in mRNA degradation, we hypothesized that AtXRN4 may contribute to the turnover of plant sRNAs.

To determine the possible role of AtXRN4 in sRNA turnover, we first compared the sRNA population differences between Col-0 WT and *xrn4* mutant plants. *ein5-1*, an EIN5 mutant that carries a “C” deletion at the fifteenth exon, was chosen for the small RNA profile analysis (Figure S3a) [20]. The AtXRN4 transcript was detected in Col-0 WT adult and Col-0 WT seedling plants (Figure S4). Moreover, the accumulation of the AtXRN4 transcript in rosette leaves was comparable to that in the rest of the tested tissues (Figure S4). Therefore, the four-week-old *ein5-1* plants grown on the soil were collected to perform further sRNA deep sequencing analyses.

With total RNA isolated from collected tissues, two sets of sRNA libraries were constructed and sequenced. After removal of adapter sequences and low-quality reads, 18,762,306, 19,905,497, 18,650,051, and 19,905,780 clean sRNA reads were obtained from Col-0 WT and *ein5-1* libraries, respectively (Figure S5a). There were 16,907,246, 18,037,364, 17,057,402, and 18,213,882 total sRNAs reads were mapped perfectly to the Arabidopsis genome for Col-0 WT and *ein5-1*, respectively. These sRNAs were clustered into 3,850,420, 4,154,868, 3,789,676, and 3,804,558 unique sRNA reads. The sample correlation matrix was evaluated with sRNA expression levels in the following color code: red represents strongly correlated and green represents weakly correlated. The matrix heatmap indicated that the sRNA libraries are highly reproducible (Figure S5b). Therefore, 4,002,644 and 3,797,117 average unique sRNAs were obtained for Col-0 WT and *ein5-1* mutant plants for further analysis, respectively.

**Figure 1:**
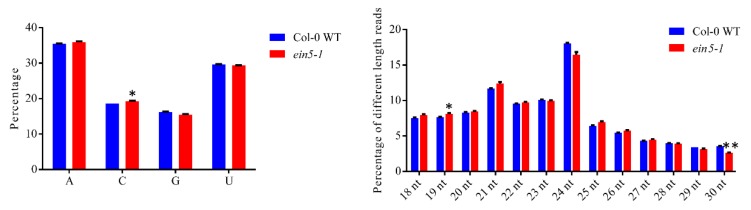
AtXRN4 mutants change the sRNA profiles. (**a**) 5′-terminal nucleotide frequency of sRNAs in Col-0 WT and *ein5-1* mutant plants. (**b**) Size distribution of sRNAs in Col-0 WT and *ein5-1* mutant plants. One star means P < 0.05, two star means P < 0.01, three star means P < 0.001, four star means P < 0.0001.

The 5’-terminal nucleotide compositions of sRNAs from different libraries were analyzed first. Uridine (U) or adenosine (A) are the dominant 5′-terminal nucleotides for both Col-0 WT and *ein5-1* libraries (Figure 1a). Moreover, the 5’-terminal nucleotide compositions of sRNAs in Col-0 WT and *ein5-1* libraries were comparable (Figure 1a). Next, the size distributions of sRNAs were analyzed. In the *ein5-1* mutants, 21nt sRNAs increased, while 24 nt sRNAs decreased, which demonstrated that the malfunction of AtXRN4 alters the length distributions of plant sRNAs (Figure 1b).

### 2.2. AtXRN4 Decreases the Accumulation of miRNA*s

Due to the increased PTGS, a previous study showed that 21 and 22nt siRNAs derived from aberrant coding transcript (ct-siRNAs) accumulated in *ein5/ski2* double mutants [26]. Indeed, the 22 nt sRNAs in *ein5-1* plants were also slightly increased over those in Col-0 WT plants (Figure 1b). This so slight increase probably because of the small percentage of ct-siRNA. However, the enhancement of 21 nt sRNAs was more abundant than that of the 22 nt sRNAs. Therefore, the accumulation of some other 21 nt sRNAs may also be increased in *ein5-1* mutant plants.

**Figure 2:**
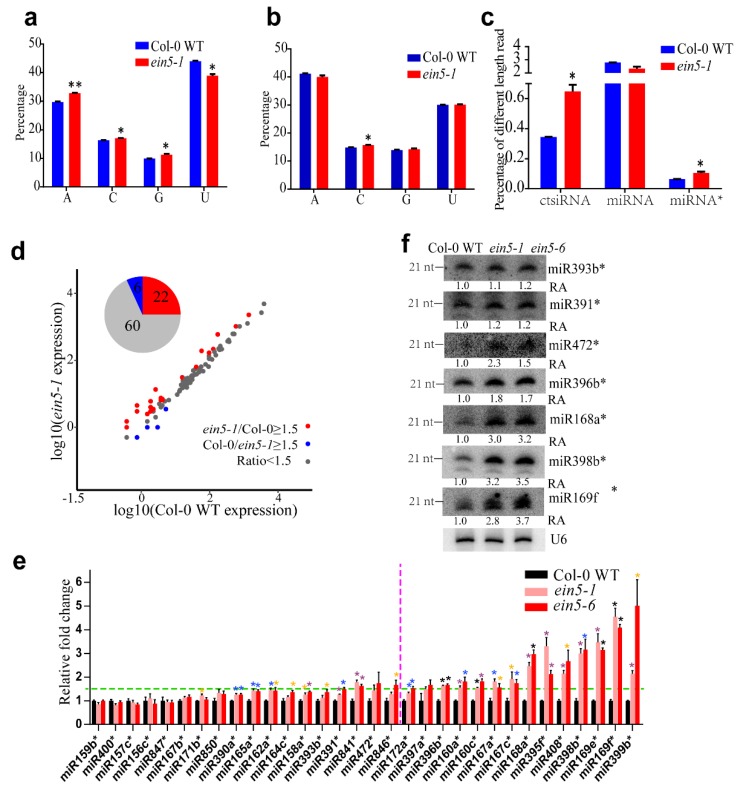
AtXRN4 decreases the accumulation of miRNA*s.(**a**) The 5′-terminal nucleotide frequency of 21 nt sRNAs in Col-0 WT and *ein5-1* mutant plants. (**b**) The 5′-terminal nucleotide frequency of 22 nt sRNAs in Col-0 WT and *ein5-1* mutant plants. (**c**) The relative accumulation of different 21 nt sRNAs in Col-0 WT and *ein5-1* mutant plants. (**d**) Mean abundance plot analysis of miRNA*s in Col-0 WT and *ein5-1* mutant plants. miRNA*s with mean reads over 2 are shown. miRNA*s with significant changes (*ein5-1*/Col-0 WT≥ 1.5 and Col-0 WT/*ein5-1*≥ 1.5) are highlighted with red and blue dots, respectively. (**e**) qRT-PCR validation of miRNA*s in different plants. The relative expression of 14 miRNA*s with significant increases (>1.5-fold) and 18miRNA*s without significant changes are shown in the right and left panels, respectively. The green line indicates the 1.5-fold change. qRT-PCR data are shown as the means ± SEM. Yellow star means P < 0.05, blue star means P < 0.01, purple star means P < 0.001, black star means P < 0.0001. Similar results were obtained in more than three biological repeats. (**f**) Northern blot was used to detect the accumulation of miRNA*s in different plants. U6 served as the loading control. Similar results were obtained in three biological repeats. Primers and probes are listed in Table S2.

To determine the 21 nt sRNAs that were enhanced in *ein5-1* mutants, the 5’-terminal nucleotide compositions of 21 nt sRNAs were analyzed. The 5’-terminal nucleotide compositions of 22 nt sRNAs were analyzed as controls. “U” and “A” were the dominant 5’-terminal nucleotides for both 21 nt and 22 nt sRNAs. The accumulation of 21 nt sRNAs containing 5’-terminal “A”, “C”, “G” was significantly increased in *ein5-1* mutant plants. The accumulation of 22nt sRNA containing 5′-terminal “C” is also increase in ein5-1 mutant plants while the 22 nt sRNAs start “A” and “G” are comparable in Col-0 WT and *ein5-1* mutant plants (Figure 2a,b). 5′-terminal preference bias is different for 21- and 22-nt sRNA, which is consistent with the published results [29,32,33]. Most miRNA*s associated with AtAGO2 have a 5’-terminal “A” [29,30]. The accumulations of miRNA*s were thus analyzed. Indeed, miRNA*s increased in *ein5-1* mutant plants (Figure 2c). Meanwhile, ct-siRNAs and miRNAs increased and decreased in *ein5-1* mutant plants, respectively. A total of 112 miRNA*s were identified in our sRNA libraries, and the accumulation of 88 of them was higher than 2 reads (or at least 1 reads in each sample) (Table S1). To further determine the profile of miRNA*s, we performed mean abundance plot analysis of these miRNA*s. Among the 88 miRNA*s analyzed, 22 miRNA*s in the *ein5-1* mutants were more than 1.5 times higher than those in Col-0 WT plants (Figure 2d and Table S1). In contrast, only 6 miRNA*s were down-regulated in *ein5-1* mutants. Therefore, AtXRN4 affects the accumulation of a subset of miRNA*s.

qRT-PCR and Northern blot assays were then conducted to further validate the role of AtXRN4 on miRNA* accumulation. *ein5-1* and *ein5-6*, another AtXRN4 mutant that contains a T-DNA insertion at the fifth exon, were used to perform qRT-PCR experiments (Figure S3a). The 14 most abundant miRNA*s that were up-regulated in *ein5-1* mutant plants (ratio > 1.5) were selected for qRT-PCR experiments (Table S1). Indeed, the accumulation of all miRNA*s tested in *ein5-1* mutant plants was significantly higher than that in Col-0 WT plants (Figure 2e, right panel). The accumulations of 18 unchanged miRNA*s (ratio < 1.5) were also determined by qRT-PCT. The accumulation of most of these miRNA*s was also increased in *ein5-1* and *ein5-6* mutants (Figure 2e, left panel). Consistent with the sRNA sequencing assays, the enhancements of most candidates in the left parts of Figure 2e were lower than those in the right parts of Figure 2e. The four most significantly increased miRNA*s, miR169f*/miR169e*, miR396a*/miR396b*, miR398b*, and miR168a* were selected for Northern blot analysis (Figure S6, miR408* is undetectable by Northern blot). The accumulations of miR393b*, miR391b*, and miR472*, the three abundant miRNA*s with weaker increases, were also tested. As shown in Figure 2f, the accumulations of miR472*, miR396b*, miR168a*, miR398b*, and miR169f* in *ein5-1* and *ein5-6* were much higher than those in Col-0 WT plants. In contrast, the accumulations of miR393b* and miR391* were slightly higher than those in control plants. We also determined the corresponding miRNA expression by qRT-PCR and Northern blot, the results showed that the accumulations of a subset of miRNAs corresponding to the highly increased miRNA*s also increased in *ein5-1* mutant plants (Figure S7). Therefore, EIN5 may alter the stability of miRNA/miRNA* duplexes. Altogether, these results indicated that AtXRN4 decreases the accumulation of a subset of miRNA*s and miRNAs.

### 2.3. AtXRN4 does not Alter theTranscription and Processing of miRNAsPrecursors

The abundance of sRNA is a balance between the biogenesis and turnover of sRNAs. In plants, the pri-miRNAs transcribed by RNA Pol II were processed by DCL1 into pre-miRNAs, which were further processed into short double stranded RNAs (miRNA/miRNA*) [34,35].

**Figure 3:**
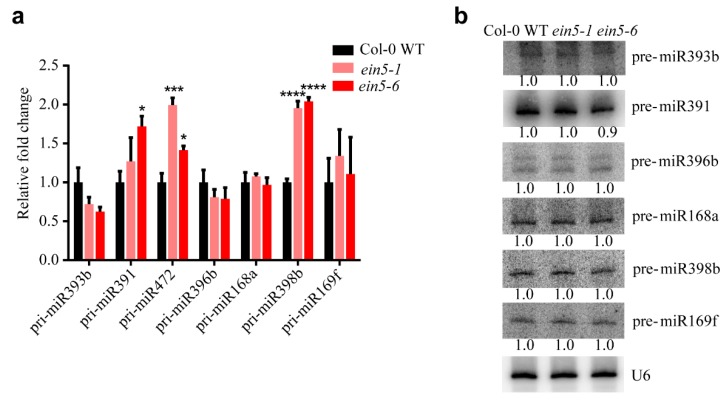
AtXRN4 does not affect the transcription and processing of miRNA*s. (**a**) qRT-PCR analysis of pri-miRNAs in Col-0 WT and *xrn4* mutant plants. Actin2 was used as an internal control. One star means P < 0.05, two star means P < 0.01, three star means P < 0.001, four star means P < 0.0001. (**b**)Northern blot analysis of the corresponding precursors of miRNA*s in Col-0 WT and *xrn4* mutant plants. U6 was used as the loading control. Probes are listed in Table S2. Similar results were obtained in three biological repeats.

The possible functions of AtXRN4 on miRNA* transcription was first evaluated. The accumulations of corresponding pri-miRNAs were detected using qRT-PCR. The accumulations of pri-miR391 in *ein5-6* and pri-miR472 and pri-miR398in *ein5-1/ein5-6* mutant plants were higher than those in Col-0 WT plants (Figure 3a). However, the accumulations of the remaining pri-miRNAs in *ein5-1* and *ein5-6* mutant plants were comparable to those in Col-0 WT plants. Portion of the pri-miRNA* tested are higher than those in the Col-0 WT plants. However, most of the accumulation of tested miRNA*s in *ein5* mutants are higher than those in the Col-0 WT plants. Similar results were obtained in *fry1* mutant [36]. Moreover, the transcription and processing of pri-miRNAs take place in nuclear, while XRN4 localizes inP-bodies [22,37]. These results argue against the general role of XRN4 on the accumulation of miRNA*s caused by the increased accumulation of pri-miRNAs. To test the possible role of AtXRN4 on miRNA* processing, we performed Northern blot analysis to detect the accumulations of corresponding pre-miRNAs. No differences in pre-miRNA accumulations were observed among Col-0 WT, *ein5-1*, and *ein5-6*, which argued against a role of AtXRN4 in miRNA* precursor processing (Figure 3b). Taken together, these results indicated that AtXRN4 decreased miRNA* accumulation downstream of the miRNA precursors processing step.

### 2.4. AtXRN2 and AtXRN3 do not Alter the Accumulations of Corresponding miRNA*s

**Figure 4:**
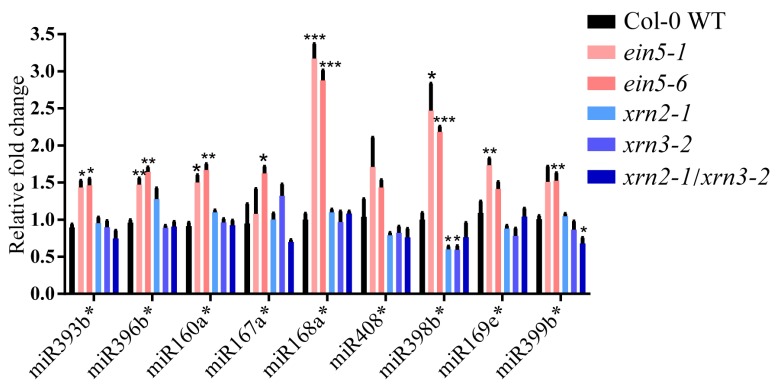
AtXRN2 and AtXRN3 do not decrease the accumulation of corresponding miRNA*s. qRT-PCR analysis of these accumulated miRNA*s in *xrn2-1*, *xrn3-2* and *xrn2-1xrn3-2* mutants seedlings. Col-0 WT, *ein5-1* and *ein5-6* were involved as control. One star means P < 0.05, two star means P < 0.01, three star means P < 0.001, four star means P < 0.0001.

AtXRN2 and AtXRN3 are also XRN homologues in Arabidopsis, the functions of them in the accumulation of these miRNA*s were then determined. Two-week-old *xrn2-1*, *xrn3-2* and *xrn2-1/xrn3-2* seedlings were collected to perform qRT-PCR. *ein5-1* and *ein5-6* seeding was involved as control and the accumulations of corresponding miRNA*s were also increased in *ein5-1* seedling. However, the accumulations of these miRNA*s were not significantly altered in *xrn2-1*, *xrn3-2* and *xrn2-1/xrn3-2* seedlings (Figure 4). We also determined the corresponding miRNA expression by qRT-PCR, the results showed that the accumulations of a subset of miRNAs corresponding to the highly increased miRNA*s also increased in *ein5-1* mutant plants (Figure S8). Taken together, these results showed that only AtXRN4 contributes the accumulation of miRNA*s. 

### 2.5. AtXRN4 Interacts with AtAGO2 in P-Bodies

After processing, miRNA/miRNA* duplexes are loaded into AtAGO, the core component of the RNA-induced silencing complex (RISC) for maturation, regulation and degradation [38]. Our results showed that AtXRN4 affects the turnover but not the transcription and processing of miRNA*s, indicating that the function of AtXRN4 may correlate with the function of AtAGO.

**Figure 5:**
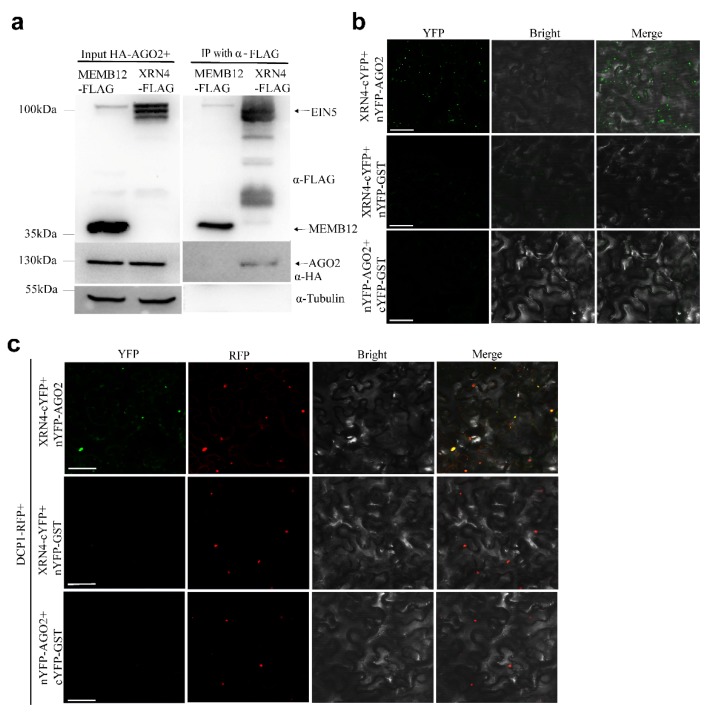
AtXRN4 interacts with AtAGO2 in P-bodies. (**a**) Co-IP experiment in *N. benthamiana* shows that AtXRN4 interacts with AtAGO2. IP was conducted using the α-FLAG antibody. Input and IP products were probed with anti-HA and anti-FLAG antibodies, respectively. (**b**) BiFC analysis of the interaction between AtXRN4 and AtAGO2. YFP images were taken 48 hpi. Scale bars, 50 μm. (**c**) AtXRN4-AtAGO2 interaction sites colocalize with DCP1, a marker protein for P-bodies. Green fluorescence indicates YFP and red fluorescence indicates DCP1-RFP. Scale bar, 50 μm.

AtAGO1 is the major AtAGO associated with miRNAs while AtAGO2 is the major AtAGO associated with miRNA*s. miRNA*s especially these with 5′ terminal A were predominantly associated with AGO2 [29,30,39]. Therefore, AtXRN4 may interact with AtAGO2 and affect miRNAs and miRNA*s turnover. Firstly, we predicted we predicted the co-expression gene of AtAGO2 at http://atted.jp/ (a plant co-expression database) and find AtXRN4 in the co-expression list. To confirm the interaction possibility, a co-immunoprecipitation (Co-IP) assay was conducted in Nicotianabenthamiana. HA-tagged AtAGO2 was co-expressed with FLAG-tagged AtXRN4 and with FLAG-tagged MEMB12 as a negative control [30]. With the α-HA antibody, HA-AtAGO2 was easily detected in AtXRN4-FLAG IP fractions but not in MEMB12-FLAG IP fractions (Figure 5a). To further confirm this interaction, a bimolecular fluorescence complementation (BiFC) assay was conducted. Both nYFP-AtAGO2 and AtXRN4-cYFP were co-expressed in N. benthamiana. In addition, nYFP-GST and cYFP-GST were co-inoculated as negative controls. Strong fluorescence was detected in nYFP-AtAGO2 and AtXRN4-cYFP co-expression plants but not in control plants (Figure 5b). These results proved the interaction between AtXRN4 and AtAGO2.

The YFP signal accumulates in punctate compartments in nYFP-AtAGO2 and AtXRN4-cYFP co-expression plants (Figure 5b). P-bodies display punctate structures in the cytoplasm, and AtXRN4has been reported to co-localize with DCP1 and DCP2, the marker proteins of P-bodies [37]. Therefore, AtXRN4 may interact with AtAGO2 in P-bodies. We thus co-inoculated nYFP-AtAGO2 and AtXRN4-cYFP with DCP1-RFP to test this possibility. The punctate structures of YFP signals largely overlapped with the RFP signals (Figure 5c). Taken together, AtXRN4 interacts with AtAGO2 in P-bodies.

## 3. Discussion

sRNA turnover is a key but poorly understood mechanism that determines the accumulation of sRNAs [3,11]. The plant exoribonuclease that conducts 5’-3’ sRNA degradation has not been identified. In this study, we found that AtXRN4, a 5’-3’ exoribonuclease in Arabidopsis, is involved in the turnover of miRNA*s. We then revealed that AtXRN4 interacts with AtAGO2, an AtAGO that primarily loads miRNA*s, in P-bodies which play a fundamental role in RNA decay. Therefore, the function of AtXRN4 in miRNA* degradation may correlate with the function of AtAGO2.

Our observations demonstrate that AtXRN4, a 5’-3’ exoribonuclease in plants, contributes to the turnover of plant miRNA*s. XRN genes play key roles in the turnover of different RNAs in eukaryotes [40,41]. In the nucleus, DmXRN2 degrades mRNAs that are decapped by LSM2-8 proteins to restrict their localization in the nucleus [42]. HsXRN2 also degrades the unprotected 5’ end of transcripts processed from mRNA to promote transcription termination [43]. In the cytoplasm, XRN1 degrades decapped mRNA in yeast, humans, flies and worms [42,44,45,46]. The role of XRN in sRNA degradation was first identified in *C. elegans*. Depletion of both CeXRN1 and CeXRN2 increased the accumulation of miRNAs or miRNA*s without altering the accumulation of pre-miRNAs [14,15]. HsXRN1, but not HsXRN2, was found to contribute to the degradation of miR382 and a subset of other miRNA*s [12]. Plants lack an XRN1 orthologue but encode three orthologs of XRN2, AtXRN2, AtXRN3, and AtXRN4 [18]. AtXRN4 degrades endogenous mRNAs and decapped transgene mRNAs [23,25]. Although the degradation of mRNA transcripts by AtXRN4 inhibits the initiation of RNA silencing of endogenous genes and transgenes [25,26], the function of AtXRN on sRNA degradation has not been determined. Here, we found that the mutation of AtXRN4 leads to the increase accumulation of a subset of miRNA*s (Figure 2) without alternations in the corresponding pri-miRNAs and pre-miRNAs (Figure 3). These results indicate that AtXRN4 is a plant 5’-3’ exoribonuclease that conducts the turnover of sRNAs.

The accumulation of rRNA-derived sRNA loaded into AtAGO1 were increased in *xrn2-1/xrn3-2* mutant plants and it leads the decreased accumulation of un-protected miRNAs [36]. However, the accumulations of test miRNA*s were not significantly altered in *xrn2-1/xrn3-2* mutant seedling (Figure 4), which is consistent with the association of miRNAs but not miRNA*s with AtAGO1 [29]. Therefore, distinct XRN genes may regulate the accumulation of different sRNAs.

The degradation of plant miRNA*s may occur in P-bodies. XRN1 co-localizes with DCP1 and DCP2, the proteins that conduct the decapping process, in P-bodies and performs mRNA degradation in yeast [47]. DCS-1, a decapping scavenger enzyme, interacts with CeXRN1 and promotes the degradation of miRNAs in *C. elegans* [48]. The human orthologue of DCS-1 is also required for the 5’-3’ exoribonuclease function of XRN2 and plays key roles in miRNA degradation [49]. As a marker protein for the 5′-3′ mRNA degradation pathway, AtXRN4 has been reported to co-localize with DCP1 in P-bodies [37]. AGO1, AGO2 and reporter mRNA that are targeted by miRNAs were also reported to localize in P-bodies [50]. P-bodies have been found to link to RNAi (RNA interference) and miRNAs mediated decay and translational repression [50,51,52,53]. Here, we proved that AtXRN4 interacts with AtAGO2, a major AtAGO that associates with miRNA*s, in P-bodies (Figure 5) [29,30,39]. ATRM2, a 3’-5’ exoribonuclease that degrades unmethylated miRNA/miRNA*s, also interacts with AtAGO1 in punctate structures in the cytoplasm [8]. These results suggest that the P-body is an organelle that degrades both mRNAs and sRNAs. AtXRN4 is likely to directly or indirectly regulate these miRNA*s in P-bodies. The interaction of AtXRN4 with AtAGO2 indicates that the function of AtXRN4 on sRNAs may correlate with AtAGO2 in P-bodies.

AtXRN4 only contributes to the turnover of a subset of miRNA*s. CeXRN1 and CeXRN2 contribute to the degradation of a subset of miRNAs in *C. elegans* [14,15,54]. The accumulation of a subset of miRNA*s with thermodynamic asymmetry also increased in Cexrn1 and Cexrn2 malfunction tissues, indicating that CeXRN1 and CeXRN2 degrade some miRNA*s [14]. HsXRN1 also only contributes to the degradation of miR382 and some other miRNAs [12]. This specific instability is derived from their 3’-terminal sequences, and a mutation in the seven 3’-terminal nucleotides of miR382 increases its stability. ATRM2, the 3′-5′ exoribonuclease in plants, also affects the accumulation of a subset of unmethylated miRNA/miRNA* duplexes [8]. However, how ATRM2 fulfills this selection bias is unknown. The accumulation of some miRNA*s is altered differently in Atxrn4 mutant plants, which indicates that AtXRN4 may also degrade a subset of miRNA*s. In Atxrn4 mutants, the up-regulated miRNA*s have different 5′ ends. Therefore, the sRNA characteristics that determine the selection bias of AtXRN4 need to be further determined.

As an important component of ethylene signal pathway, AtXRN4 interacts with EIN2 to targets *EBF1* 3′ UTR to cytoplasmic P-body [22]. AtXRN4 mutants showed ethylene insensitive phenotype [20]. Ethylene has been found to be key factors for fruit ripening, seed germination, sexual determination of flower, root nodulation, and response to pathogens and stresses [55]. The effect of AtXRN4 malfunction on miRNA pathway may be the result on ethylene signaling pathway. This possibility cannot be ruled out and deserves further study.

## 4. Materials and Methods

### 4.1. Plant Materials and Growth Conditions

Plants were grown in a green house at 22 °C, 70% humidity and a 12-h light/12-h dark photoperiod. *ein5-1* [19] and *ein5-6* [20] mutant plants have been isolated by Dr. Joseph R. Ecker’s group previously. These seeds are kindly gifts from Dr. Hongwei Guo and Dr. Menji Cao. *xrn2-1*, *xrn3-2* and *xrn2-1/xrn3-2* seeds are kindly gifts from Dr. Beixin Mo [36]. The *ein5-1, ein5-6*, *xrn2-1* and *xrn3-2* mutant seeds are also available on TAIR database (www.arabidopsis.org).

### 4.2. RNA Extraction and sRNASequencing

Total RNA isolation and library construction were performed as described previously [33]. Briefly, four-week-old plants were collected, and RNA was isolated with TRIzol (Invitrogen, Carlsbad, CA, USA,) according to the manufacturer’s instructions. Approximately 1 g rosette leaves were ground in liquid nitrogen and mixed with 10 mL TRIzol for RNA isolation. After thoroughly mixing, 1/2 volume chloroform was added. The solution was then vortexed thoroughly and put on ice for 5min. Then it was centrifuge at 12000rpm and 4 °C for 15 min. The supernatant was mixed with 2.2-fold volume of ethanol and stored at −20 °C overnight. The mix was then centrifuge at 12000rpm and 4 °C for 15 min. The pellet was washed with 75% ethanol and finally dissolved with RNase-free H2O. The concentration and quality of the isolated RNA were determined by a NanoDrop spectrophotometer (Thermo Scientific, Waltham, MA, USA) and gel electrophoresis, respectively. Total RNA (20 µg) was used for sRNA library construction with the Illumina TruSeq sRNA Library Preparation Kit (Illumina, San Diego, CA, USA). 15–30nt sRNAs were cut from the gel for library construction. TwosRNA libraries were constructed for both Col-0 WT and *ein5-1* samples. sRNA sequencing was performed with an Illumina HiSeq 2500 instrument.

### 4.3. sRNA Sequencing Analysis

We used fast for removing adapter and filtering low-quality reads [56]. Bowtie was used to map reads to Arabidopsis genome with specific parameters (bowtie -f -v 1 -p 10 -a -m 1 --best --strata) [57]. Correlation was calculated using Euclidean’s distance matrix using PtR program in Trinity package [58]. Reads count was also calculated by BEDTOOLS with unique mapping reads. BLAT was used to find the best hit of miRNA* in the genome [59]. BEDTOOLS was used to intersect the locus between miRNA and miRNA* in order to make sure the miRNA-miRNA* pair in the same transcript [60]. Differential gene expression analyses of replicated count data were carried out using the R package EdgeR [61]. The reads count of miRNA*s from different samples were normalized by the number of total tRNA reads as performed in previous study [62].

### 4.4. qRT-PCR

For sRNA qRT-PCR, 1 µg of total RNA was digested with DNase I (Takara, Dalian, China, and poly(A) was added to the 3′ end by E. coli poly(A) polymerase (NEB). The first-strand cDNAs were transcribed by the Moloney murine leukemia virus (M-MuLV) reverse transcriptase (NEB). qRT-PCR was performed in a 96-well plate with TB GreenTM Premix Ex TaqTM (TIi RNase H Plus) (Takara, Dalian, China). The 10 µl PCRcontained 5 µl 2x TB Green™ Premix Ex Taq™, 2 µl cDNA template (diluted 1/10-fold before use) and 200 nM primers. Every sRNA forward primers were listed in Table S2 and all reverse primers are 3′ race inner listed in Table S2. The PCR was performed as follows: 95 °C for 30 sec and 40 cycles of 95 °C for 5 sec and 60 °C for 30 sec. U6 was used as an endogenous control.

For mRNA qRT-PCR, 1 µg of total RNA was used for reverse transcription according to the manufacturer’s instructions with the PrimeScript™ RT reagent Kit with a gDNA Eraser (Takara, Dalian, China) and the random primer were used in the kit. The PCR was the same as the sRNA qRT-PCR process except for primers which were also listed in Table S2. Actin2 was used as the control. The relative fold change in the expression levels were calculated using the 2^−ΔΔCt^ method. All reactions were carried out in two technical repeats and four biological repeats.

### 4.5. Northern Blot

A total of 60 µg total RNA was loaded onto a 14% denaturing urea-polyacrylamide gel and run with 0.5x TBE at 150V. The gel was transferred to Amersham HybondTM-NX with 14A overnight. Chemical cross-linking buffer was prepared as follows: 0.373 g N-(3-Dimethylaminopropyl)-N′-ethylcarbodiimide hydrochloride (Sigma-Aldrich, E7550, St. Louis, Missouri, MI, USA), 3 drops of 1 M HCl, 121 µl 1-Methylimidazole (Sigma, M50834), and 12 mL DEPC-treated ddH2O. Membrane was putted on a plate which has two layers of filter paper that was soaked in cross-linking buffer, then the plate was covered by plastic film andincubatedat60 °C for 2 h and at 85 °C for another2 h. The probes were labeled withγ-P32 ATP by T4-polynucleotide kinase (NEB). Pre-miRNA Northern blot probes are the same as miRNA Northern blot probes. The membrane was pre-incubated with PerfectHyb™ Plus Hybridization Buffer liquid (Sigma) for 30min, then the labeled probe was added and incubated for 12h. After that, the membrane was wash with buffer contains 2x SSC and 0.025% SDS for four times. Signal was collected with phosphor screen and scanned with typhoon. Similar results were obtained from three biological repeats.

### 4.6. Plasmid Construction

To perform the Co-IP assay, XRN4, MEMB12 and AtAGO2 were cloned into the pENTR vector (Invitrogen, Carlsbad, CA, USA, K2420-020) and then recombined into the Gateway destination vectors pEarlyGate202 (pEG202), pFH, and pMDC32 [63,64,65] by the LR reaction with the LR enzyme (Invitrogen, Carlsbad, CA, USA, 11791-020).

To conduct the BiFC assay, the GST fragment was cloned into the pENTR vector. GST, XRN4, and AtAGO2 fragments were then cloned into pSITE-cEYFP or pSITE-nEYFP vector by the LR reaction.

For the co-localization assay, the DCP1 fragment was cloned into the pENTR vector. The fragment was then cloned into pEarlyGate102 (pEG102) by the LR reaction.

### 4.7. BiFC and Colocalization Assay

XRN4-pSITE-cEYFP-AtXRN4, pSITE-nEYFP-AtAGO2, pSITE-nEYFP-GST and pSITE-cEYFP-GST constructs were transformed into the Argobacterium strain GV3101. Argobacterium were then infiltrated into N. benthamiana with an OD600nm = 1.0 as described before [66]. Fluorescence was determined at 72hourspost-inoculation(hpi) with a Zeiss LSM-710 confocal microscope (Carl Zeiss, Thornwood, NY, USA). YFP fluorescence was excited at 514 nm.

For the co-localization analysis, XRN4-pSITE-cEYFP-AtXRN4 and pSITE-nEYFP-AtAGO2 were co-inoculated with pEG102-DCP1-RFP in N. benthamiana. YFP and RFP fluorescence were excited at 514 nm and 561 nm, respectively. Similar results were obtained from more than three biological replicates.

### 4.8. Co-IP

pEG202-AtXRN4 and pMDC32-AtAGO2 were co-infiltrated into four-week-old N. benthamiana. Samples were collected at 72 hpi. MEMB12-pFH was used as a negative control. Leaves were ground with liquid nitrogen and extracted with 15 mL extraction buffer (20 mM Tris-HCl (pH 7.5), 300 mM NaCl, 5 mM MgCl2, 5 mM dithiothreitol [DTT], 0.5% TWEEN-20, and 1 tablet of complete protease inhibitor (Roche)). After centrifugation (5000 rpm, 15 min), the supernatants were incubated with FLAG beads (Sigma Aldrich, USA, A2220) for 4 h. Then, the beads were washed three times with washing buffer (150 mM NaCl, 5 mM DTT, 5 mM MgCl2, 0.3% Triton X-100, 20 mM Tris-HCl (pH 7.5), and 1 pellet per 50 mL Complete EDTA-free protease inhibitor (Roche)). Monoclonal anti-HA-HRP (Santa Cruz Biotechnology, sc7392), monoclonal anti-FLAG-HRP (Sigma, A8592) and monoclonal anti-α-tubulin (Sigma, T6074) antibodies were used for Western blot. Similar results were obtained from three biological replicates

## 5. Conclusions

Taken together, our observations indicate that AtXRN4 contributes to the turnover of sRNAs. AtXRN4 interacts with AtAGO2 and may degrade sRNAs in P-bodies. Our results provide new insight into plant sRNAs homeostasis and further studies needed to be performed to determine its function mechanism.

## Data Availability

The sRNA deep sequencing data has been deposited in the Sequence Read Archive (SRA) database with Bioproject accession number PRJNA587899.

## Supplementary Materials

The following are available online at https://www.mdpi.com/2223-7747/9/3/362/s1, Table S1: miRNA*s in Col-0 WT and *ein5-1* plants. Table S2: Primers and probes used in the study. Figure S1: Phylogenetic tree analysis of XRN homolog proteins in 12 species. Figure S2: Alignment of the A. thaliana XRN4 protein with orthologs from Ce, C. elegans; Hs, H. sapiens; Sc, S. cerevisiae. Figure S3. *xrn4* mutant plants display mild developmental phenotype similar to observed before. Figure S4: The relative expression of AtXRN4 in different tissues and stages in Col-0 WT plants. Figure S5: Summary of sRNA sequencing data and heatmap of the Pearson correlation between the expression levels of sRNA in Col-0 WT and *ein5-1* mutant plants. Figure S6: Difference analysis of miRNA* accumulations between two plants. Figure S7: miRNA expression level. Figure S8: miRNA expression level in two-week seedling of *ein5, xrn2* and *xrn3* mutants.
