# Supplementary material for: AtXRN4 Affects the Turnover of Chosen miRNA*s in Arabidopsis
Source: Plants (Basel). 2020 Mar 13;9(3):362. doi: 10.3390/plants9030362 (PMC7154835; doi:10.3390/plants9030362)
Supplement: Supplementary file 1 [file plants-09-00362-s001.pdf]

AtXRN4 affects the turnover of chosen miRNA*s in *Arabidopsis*

Yan Liu[1,2,†], Wenrui Gao[1,3†], Shuangyang Wu[1,2†], Lu Lu[1], Yaqiu Chen[1,4], Junliang Guo[1,5], Shuzhen Men[3], Xiaoming Zhang[1,2*]

[1]State Key Laboratory of Integrated Management of Pest Insects and Rodents, Institute of Zoology, Chinese Academy of Sciences, Beijing 100101, China

[2]CAS Center for Excellence in Biotic Interactions, University of Chinese Academy of Sciences, Beijing 100049, China

[3]Department of Plant Biology and Ecology, College of Life Sciences, Nankai University and Tianjin Key Laboratory of Protein Science, Tianjin 300071, China

[4]Henan Normal University, Department of Life Sciences, Xinxiang, Henan 453007, China

[5]Institute of Physical Science and Information Technology, Anhui University, He fei, Anhui 230601, China

† These authors contributed equally to this work.

* Correspondence: zhangxm@ioz.ac.cn

Supplemental Figure 1. Phylogenetic tree analysis of XRN homolog proteins in 12 species. The sequences were aligned using CLUSTALW and a tree constructed using maximum likelihood method with bootstrap1000 replications. The resulting tree was rooted using the *A. thaliana* SDN1 as outgroup. *C. elegans*, *Caenorhabditis elegans*;

D. melanogaster, *Drosophila melanogaster*; *H. sapiens*, *Homo sapiens*; *M, musculus*, *Mus musculus*; *S. pombe*, *Schizosaccharomyces pombe*; *C. albicans, Candida albicans*; *S. cerevisiae*, *Saccharomyces cerevisiae*; *K. lactis*, *Kluyveromyces lactis*; *A. thaliana*, *Arabidopsis thaliana*; *N. benthamiana*, *Nicotiana benthamiana*; *O. sativa*, *Oryza sativa*; *Z. mays*, *Zea mays*.

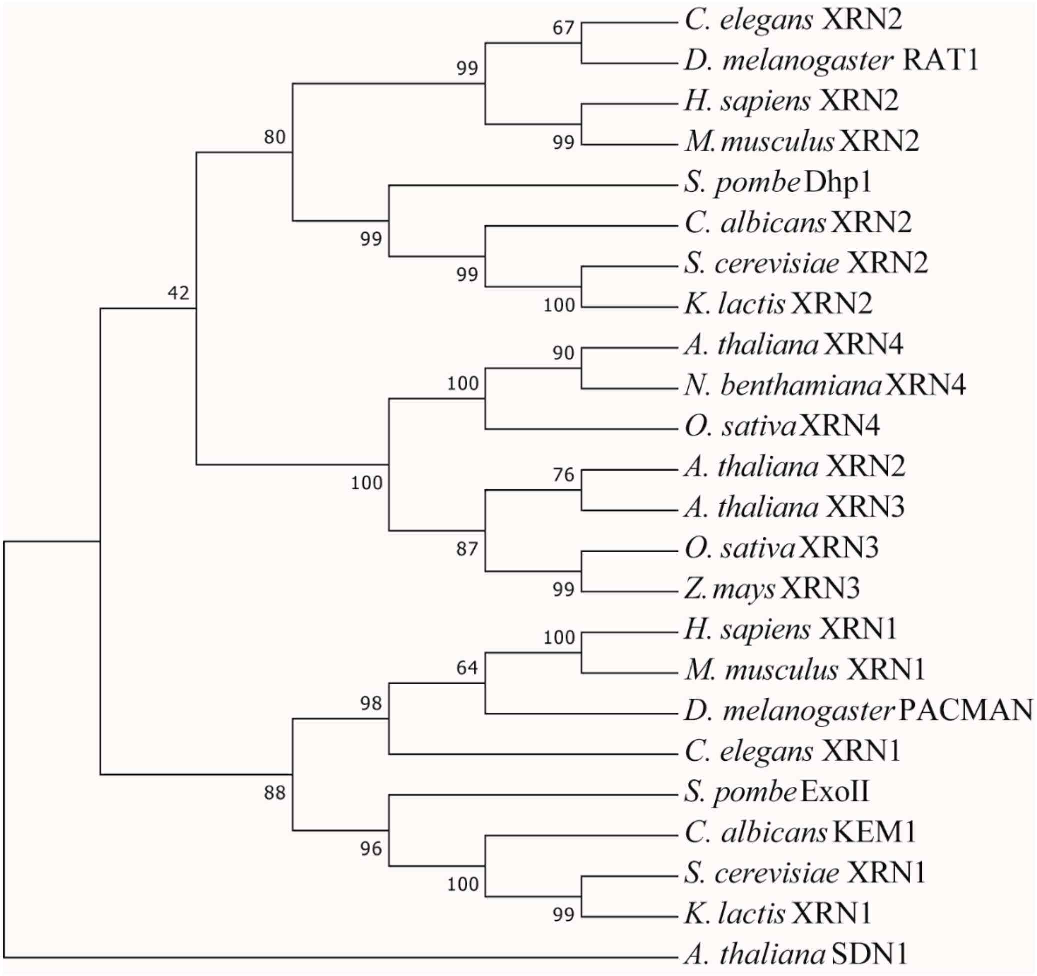

Supplement Figure 2. Alignment of the *A. thaliana* XRN4 protein with orthologs from Ce, *C. elegans;* Hs, *H. sapiens;* Sc, *S. cerevisiae.* Red star represents amino acids in the enzyme active site. CR1 and CR2 represent two highly conserved regions. Only XRN1 subfamily contains C-terminal extension. D1-D4 represent four domain

in XRN. There location was mainly marked as HsXRN1 location[1].

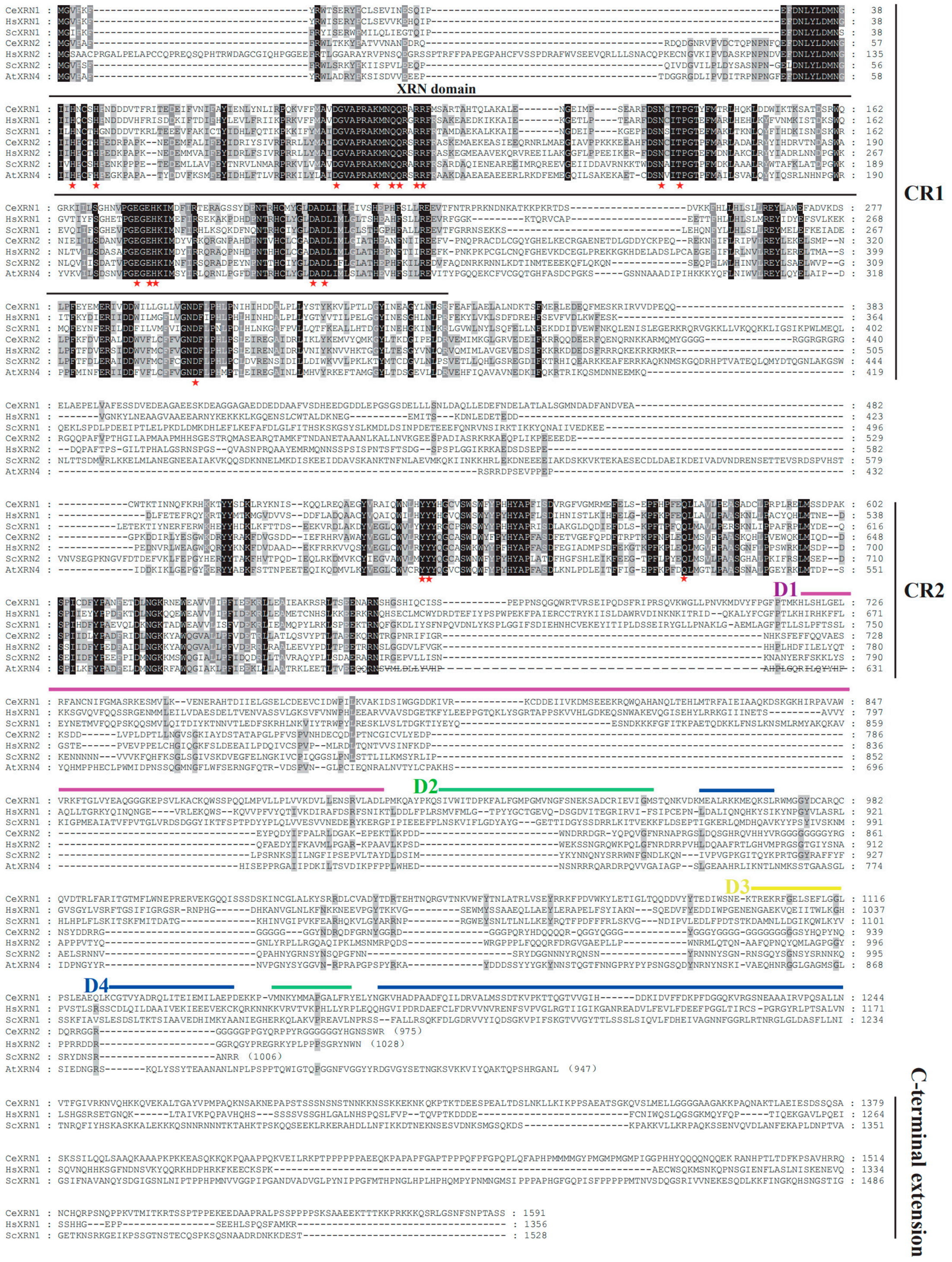

Supplement Figure 3. *xrn4* mutant plants display mild developmental phenotype similar to observed before. (a) AtXRN4 gene structure and two xrn4 mutants used in this study. (b) mutant plants show serrated leaves. (c) Mutant plants show abnormal inflorescence stems. (d) Mutant plants show delayed flowering phenotype.

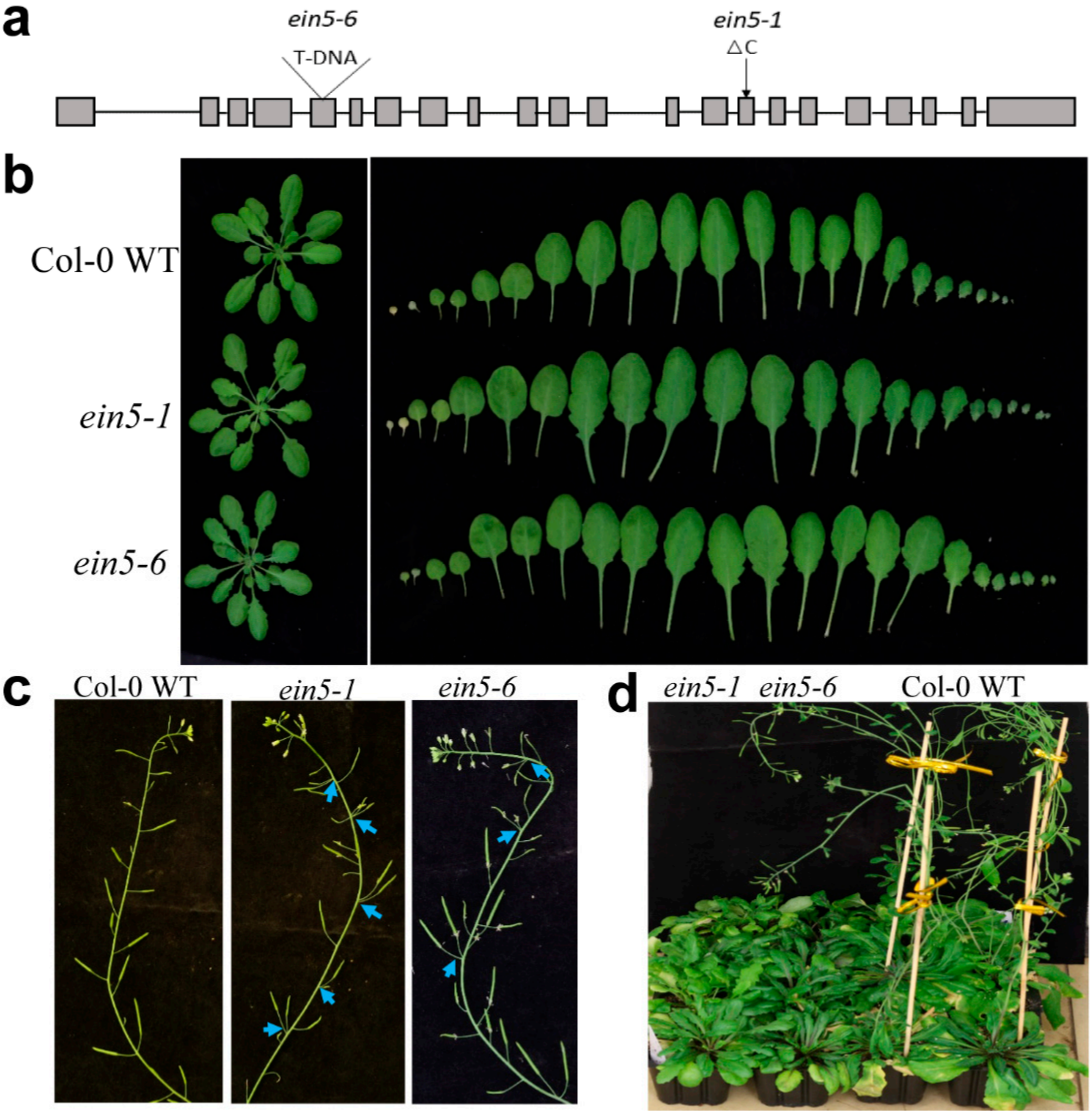

Supplement Figure 4. The relative expression of *AtXRN4* in different tissues and stages.

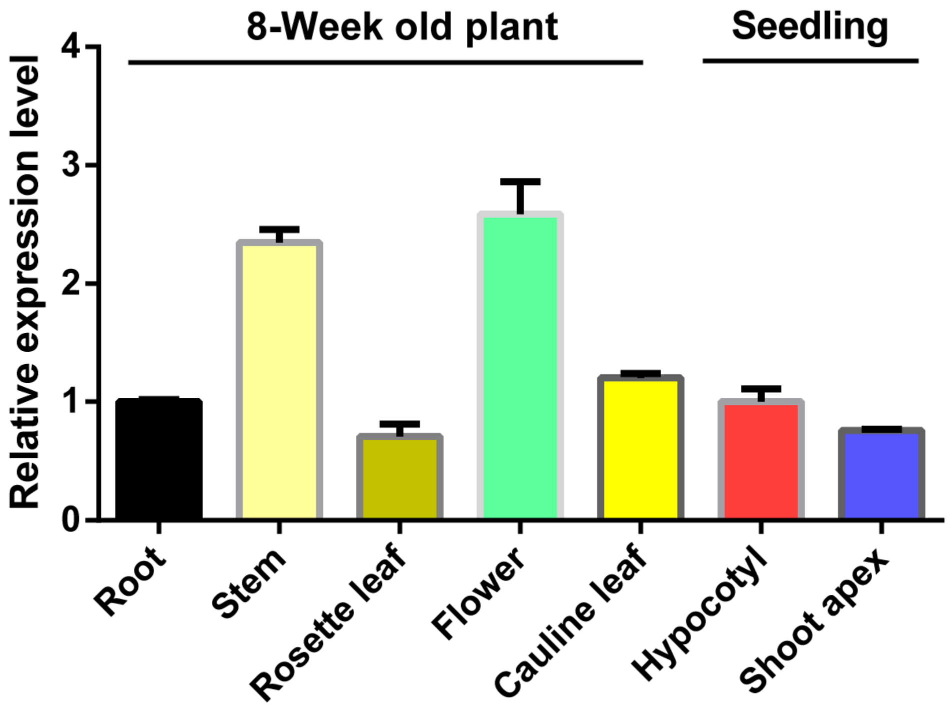

Supplement Figure 5. Summary of sRNA sequencing data and heatmap of the Pearson correlation between the expression levels of sRNA in Col-0 WT and ein5-1 mutant. (a) Summary of sRNA sequencing data in Col-0 WT and *ein5-1* mutant plants. #1 and #2 mean two different libraries. (b) Heatmap of the Pearson correlation between the expression levels of sRNA in Col-0 WT and *ein5-1* mutant plants. Red color represents higher correlation while green color represents lower correlation.

a

Sample	Total reads	Reads after filtering	Total mapped reads	Unique mapped reads	Mapping rate
Col-0 WT#1	21,486,298	18,762,306	16,907,246	3,850,420	90.11%
Col-0 WT#2	22,755,000	19,905,497	18,037,364	4,154,868	90.61%
ein5-1#1	21,420,255	18,650,051	17,057,402	3,789,676	91.46%
ein5-1#2	23,073,236	19,905,780	18,213,882	3,804,558	91.50%

b

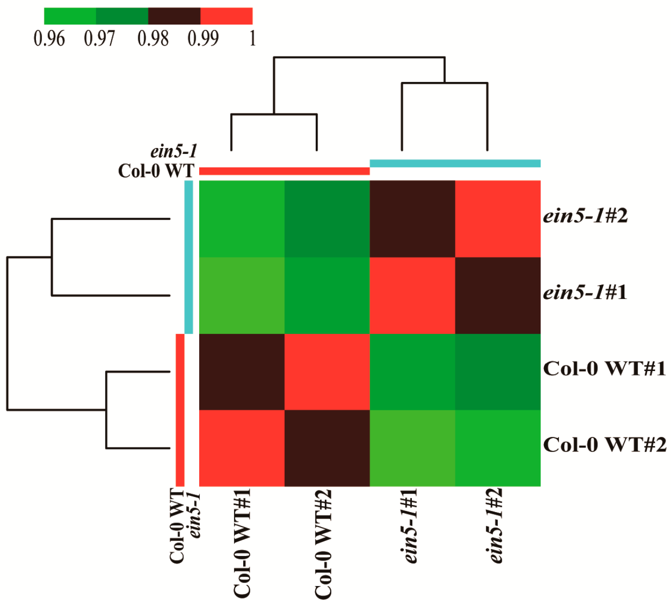

Supplement Figure 6. Difference analysis of miRNA* accumulations between two plants. Red dots and blue dots represent the significant up- and down-regulated miRNA*s respectively (P value<0.01 and fold change>1.2). Gray dots mean miRNA*s without statistical difference between Col-0 WT and *ein5-1* plants.

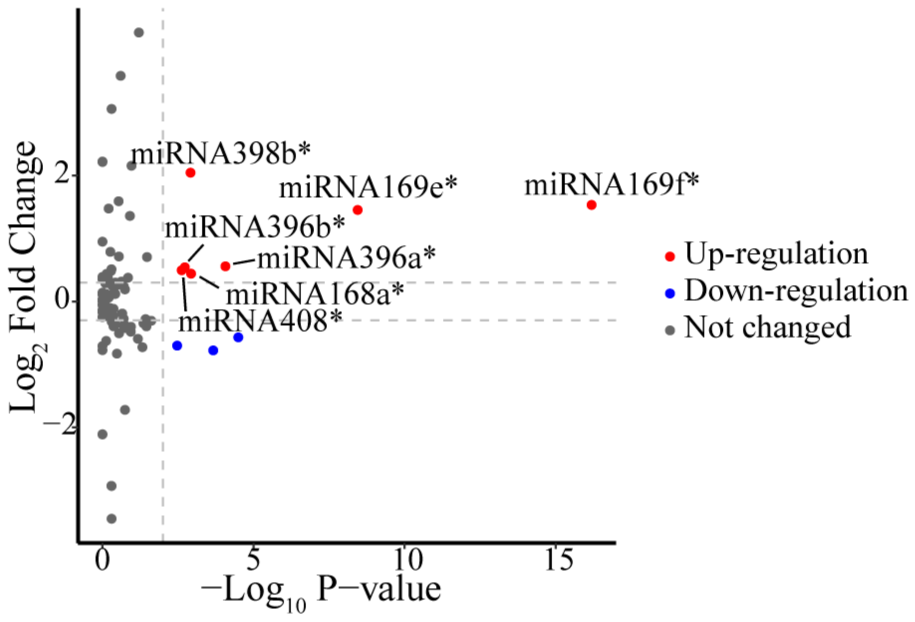

Supplement Figure 7. miRNA expression level. a) miRNA expression level by qRT-PCR. qRT-PCR data are shown as the means ± SEM. Yellow star means P<0.05, blue star means P<0.01, purple star means P<0.001, black star means P<0.0001. b) miRNA expression level by northen blot. U6 served as the loading control.

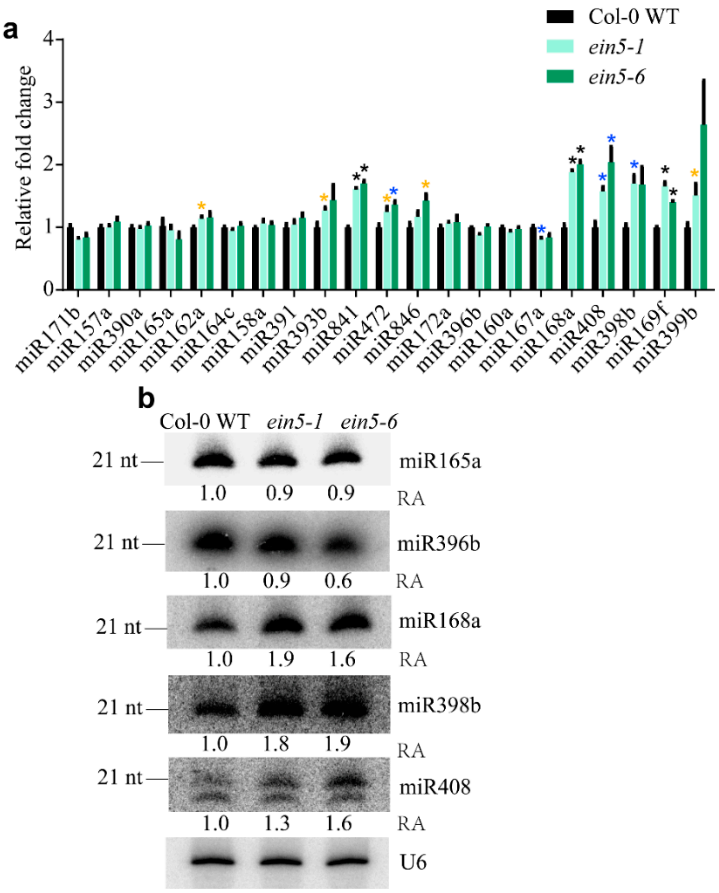

Supplement Figure 8. miRNA expression level in two-week seedling of *ein5, xrn2* and *xrn3* mutants. qRT-PCR data are shown as the means ± SEM. One star means P<0.05, two star means P<0.01.

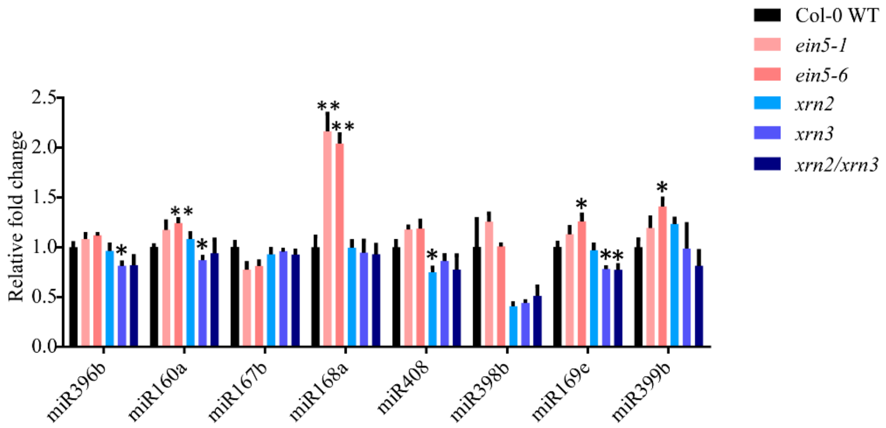

Supplement Table 1. miRNA*s in Col-0 WT and *ein5-1*

	miRNA* abundance				*ein5-1* vs. Col-0 WT		
miRNA*s	Col-0 WT#1	Col-0 WT#2	*ein5-1*#1	*ein5-1*#2	(Ratio⩾⩽1.5)	log2foldchange	P-value
ath-miR159a	1	0	2	0	up	0.951074334	1
ath-miR160c	39	42	70	57	up	0.378897315	0.14283
ath-miR163	3	5	8	5	up	0.379431954	0.663727
ath-miR166f	1	3	3	3	up	0.390826657	1
ath-miR167c	13	19	25	37	up	0.706786351	0.033551
ath-miR168a	1435	1360	2387	2179	up	0.44247992	0.001165
ath-miR169e	71	45	166	217	up	1.455097994	3.59E-09
ath-miR169f	149	197	527	663	up	1.535058635	6.50E-17
ath-miR169h	0	4	4	8	up	1.360294973	0.12332
ath-miR172a	5	3	6	8	up	0.484079313	0.523608
ath-miR395f	2	1	4	1	up	0.440878068	0.726596
ath-miR396a	594	580	1005	1068	up	0.55964014	8.53E-05
ath-miR396b	92	101	167	170	up	0.544353785	0.00188
ath-miR397a	1	0	0	3	up	1.477228341	0.625023
ath-miR398b	2	3	18	9	up	2.047425192	0.001218
ath-miR399b	1	1	5	4	up	2.157719783	0.109429
ath-miR404	1	3	4	4	up	0.78948553	0.548897
ath-miR408	116	140	229	204	up	0.496677825	0.002389
ath-miR825	4	4	6	9	up	0.715522284	0.286442
ath-miR830	3	2	5	2	up	0.354055832	1
ath-miR845a	2	5	8	5	up	0.513126851	0.503577
ath-miR849	1	1	5	1	up	1.593206209	0.292224
ath-miR156a	18	23	26	21	no	-0.052498634	1
ath-miR156b	40	43	46	31	no	-0.36805635	0.189438
ath-miR156c	132	144	190	172	no	0.130710006	0.431152
ath-miR156d	14	18	19	11	no	-0.368930797	0.374606
ath-miR157c	400	321	423	385	no	-0.106027472	0.5401
ath-miR158a	108	127	101	112	no	-0.395459557	0.035075
ath-miR159b	94	100	130	94	no	-0.054350621	0.802586
ath-miR160a	14	20	28	21	no	0.300941163	0.374767
ath-miR160b	23	17	34	22	no	0.252164074	0.478398
ath-miR162a	17	10	9	15	no	-0.394511035	0.43086
ath-miR164b	38	35	49	26	no	-0.228234251	0.434323
ath-miR164c	35	28	35	31	no	-0.201958269	0.516963
ath-miR165a	206	204	286	248	no	0.11682598	0.465227
ath-miR165b	22	24	29	30	no	0.099040649	0.775038
ath-miR166c	1	0	0	1	no	0.114399848	1
ath-miR166d	0	1	0	1	no	0.11482522	1
ath-miR166e	188	180	187	258	no	0.0198532	0.928338
ath-miR167a	33	42	52	56	no	0.259924729	0.338046
ath-miR167b	33	26	32	37	no	-0.04812337	0.932133
ath-miR167d	16	14	19	18	no	0.070946825	0.902257
ath-miR168b	779	726	1036	1028	no	0.192375864	0.180094
ath-miR169a	18	26	31	26	no	0.122248143	0.773999
ath-miR169b	1	0	1	0	no	0.114394081	1
ath-miR171b	13	10	8	11	no	-0.505516911	0.282712
ath-miR171c	18	22	20	26	no	-0.056505416	0.914324
ath-miR172b	3	2	1	3	no	-0.625759595	0.753944
ath-miR173	3783	3992	4839	4878	no	0.062469152	0.621251
ath-miR1888a	25	11	23	14	no	-0.212103962	0.705906
ath-miR2111b	1	2	2	1	no	-0.246965792	1
ath-miR319a	3	5	4	4	no	-0.16897888	1
ath-miR319b	23	21	20	19	no	-0.422580047	0.18802
ath-miR390a	146	158	160	126	no	-0.346045453	0.04398
ath-miR390b	28	34	43	28	no	-0.060927476	0.935977
ath-miR391	358	389	379	344	no	-0.305595199	0.024205
ath-miR393a	82	105	74	56	no	-0.776563699	0.000216
ath-miR393b	3340	3198	2827	2471	no	-0.569379382	3.20E-05
ath-miR398a	453	598	592	632	no	-0.031028096	0.846341
ath-miR399a	307	364	464	279	no	-0.119605471	0.547474
ath-miR399c	7	6	5	8	no	-0.235029378	0.845081
ath-miR400	1296	1261	1335	1195	no	-0.279604025	0.037602
ath-miR402	2	2	5	1	no	0.140295296	1
ath-miR403	208	244	233	199	no	-0.323838298	0.035341
ath-miR472	429	524	621	615	no	0.120135934	0.377651
ath-miR773a	6	4	6	6	no	0.03120138	1
ath-miR775	24	30	24	19	no	-0.59295239	0.06728
ath-miR781a	33	29	21	32	no	-0.480094274	0.115565
ath-miR822	45	57	51	56	no	-0.184720866	0.483818
ath-miR823	74	78	84	67	no	-0.273948085	0.199107
ath-miR838	3	4	5	5	no	0.290707493	0.803667
ath-miR840	44	54	41	47	no	-0.401055602	0.114186
ath-miR841a	45	68	65	64	no	-0.065782499	0.83445
ath-miR843	61	68	56	39	no	-0.700901415	0.003371
ath-miR844	19	24	16	15	no	-0.726171808	0.047643
ath-miR846	82	109	144	125	no	0.23774742	0.196174
ath-miR847	187	207	216	197	no	-0.1908514	0.22106
ath-miR848	23	23	29	24	no	-0.055747219	0.922925
ath-miR850	363	347	398	388	no	-0.115809308	0.432848
ath-miR853	28	38	52	47	no	0.317673866	0.236231
ath-miR868	5	3	6	3	no	-0.138150834	1
ath-miR870	12	15	13	13	no	-0.318980429	0.496974
ath-miR164a	4	7	4	3	down	-0.828132586	0.332433
ath-miR166a	2	1	1	1	down	-0.773369987	1
ath-miR394b	2	2	1	1	down	-0.773236248	1
ath-miR416	4	2	1	1	down	-1.721813557	0.179748
ath-miR776	2	0	0	1	down	-0.707945698	1
ath-miR842	1	1	1	0	down	-0.70770779	1
ath-miR158b	0	0	1	1		3.06059133	0.500015
ath-miR169d	0	0	1	0		2.217337196	1
ath-miR395a	0	0	1	0		2.22390531	1
ath-miR395d	0	0	1	0		2.220506565	1
ath-miR397b	0	0	0	1		2.224585068	1
ath-miR399f	0	0	2	3		4.274850916	0.062519
ath-miR771	0	0	1	0		2.22390531	1
ath-miR777	0	0	0	1		2.223911077	1
ath-miR859	0	0	0	1		2.223911077	1
ath-miR860	0	0	0	3		3.58673957	0.250023
ath-miR156f	1	1	0	0		-2.9316131	0.500015
ath-miR166g	0	1	0	0		-2.109085858	1
ath-miR169i	0	1	0	0		-2.109085858	1
ath-miR169j	0	1	0	0		-2.109085858	1
ath-miR170	0	1	0	0		-2.109085858	1
ath-miR2111a	1	2	0	0		-3.452041037	0.500015
ath-miR319c	0	1	0	0		-2.109085858	1
ath-miR399d	0	2	0	0		-2.92876757	0.50603
ath-miR401	1	0	0	0		-2.112497999	1
ath-miR426	2	1	0	0		-3.452213107	0.500015
ath-miR774a	2	0	0	0		-2.931856775	0.500015
ath-miR827	2	0	0	0		-3.452385156	0.500015
ath-miR839	0	1	0	0		-2.109085858	1
ath-miR852	0	2	0	0		-2.931082534	0.500661

Supplement Table 2. Primers and probes used in the study.

Name	Sequence (5'>3')	Purpose
U6 probe	AGGGGCCATGCTAATCTTCTC	RNA blotting
miR391*probe	GCTACGTAGGAGAGATACCGT	RNA blotting
miR393b*probe	AATCCAAAGA GATCGCATGAT	RNA blotting
miR472*probe	GGTATGGGCGGAGTAGGAAAAA	RNA blotting
miR396b*probe	TTTCCCACAG CTTTCTTGAGC	RNA blotting
miR168a*probe	ATTCAGTTGATGCAAGGCGGG	RNA blotting
miR169f*probe	GCAGAGCCAAGGTCAACTTGC	RNA blotting
miR398b*probe	GTGTGTTCTCATATCAACCCT	RNA blotting
miR159b*RTF	GAGCTCCTTGAAGTTCAATGG	qRT-PCR
miR171b*RTF	AGATATTAGTGCGGTTCAATC	qRT-PCR
miR162a*RTF	GGAGGCAGCGGTTCATCGATC	qRT-PCR
miR168a*RTF	CCCGCCTTGCATCAACTGAAT	qRT-PCR
miR398b*RTF	AGGGTTGATATGAGAACACAC	qRT-PCR
miR393b*RTF	ATCATGCGATCTCTTTGGATT	qRT-PCR
miR472*RTF	ATGGTCGAAGTAGGCAAAATC	qRT-PCR
miR160a*RTF	GCGTATGAGGAGCCATGCATA	qRT-PCR
miR164c*RTF	CACGTGTTCTACTACTCCAAC	qRT-PCR
miR158a*RTF	CTTTGTCTACAATTTTGGAAA	qRT-PCR
miR165a*RTF	GGAATGTTGTCTGGATCGAGG	qRT-PCR
miR408*RTF	ACAGGGAACAAGCAGAGCATG	qRT-PCR
miR391*RTF	ACGGTATCTCTCCTACGTAGC	qRT-PCR
miR169f*RTF	GCAAGTTGACCTTGGCTCTGC	qRT-PCR
miR841*RTF	CAATTTCTAGTGGGTCGTATT	qRT-PCR
miR390a*RTF	CGCTATCCATCCTGAGTTTCA	qRT-PCR
miR396b*RTF	GCTCAAGAAAGCTGTGGGAAA	qRT-PCR
miR397a*RTF	CCAGCGTTGCGCTCAATTATG	qRT-PCR

miR172a*RTF	GTGGCATCATCAAGATTCACA	qRT-PCR
miR400*RTF	GACTTATGATAATCTCATGAA	qRT-PCR
miR157c*RTF	GCTCTCTATACTTCTGTCACC	qRT-PCR
miR847*RTF	TCTTGATGAAGAGGAATGGGA	qRT-PCR
miR156c*RTF	GCTCTCTATACTTCTGTCACC	qRT-PCR
miR846*RTF	TTCAAGGACTTCTATTCAGAA	qRT-PCR
miR160c*RTF	GCGTACAAGGAGTCAAGCATG	qRT-PCR
miR167b*RTF	GGTCATGCTCTGACAGCCTCACT	qRT-PCR
miR167a*RTF	GATCATGTTCGCAGTTTCACC	qRT-PCR
miR167c*RTF	AGGTCATGCTGGTAGTTTCAC	qRT-PCR
miR395f*RTF	GTTCCCTTAAACGCTTCATTG	qRT-PCR
miR169e*RTF	GCAAGTTGACTTTGGCTCTGT	qRT-PCR
miR399b*RTF	GGGCGCCTCTCCATTGGCAGG	qRT-PCR
U6 RTF	GGGGACATCCGATAAAATT	qRT-PCR
U6 RTR	TGTGCGTGTCATCCTTGC	qRT-PCR
3'RACE_Inner	CGCGGATCCTCCACTAGTGATTTCACTATAGG	qRT-PCR
pri-miR393bF	GAGAGAGTTCTTCACAGCAA	qRT-PCR
pri-miR393bR	CATGATCCGGAAAAGTAAGC	qRT-PCR
pri-miR391F	TATATGGATGAGGTGATACT	qRT-PCR
pri-miR391R	TCTCCGTATTAAGATGCATA	qRT-PCR
pri-miR472F	CTCATCAAAGATGGATTCGGGA	qRT-PCR
pri-miR472R	TGTATGTATGGTATGGGCGGAG	qRT-PCR
pri-miR396bF	TGAAGATCCTGGTCATACTTTTC	qRT-PCR
pri-miR396bR	GAATCAATGGAGTAAAACCCTG	qRT-PCR
pri-miR168aF	ATTCGCTTGGTGCAGGTCG	qRT-PCR
pri-miR168aR	TCCAGATCTGATAGGATTTACGAGT	qRT-PCR
pri-miR398bF	TGGATCTCGACAGGGTTGAT	qRT-PCR
pri-miR398bR	GCCTTGATAAAAGATGGACGG	qRT-PCR

pri-miR169fF	TGGGAGTCTTTGGTTGTATC	qRT-PCR
pri-miR169fR	TCTGGAATCTAACATGGAAG	qRT-PCR
XRN4-F	ACAAGCCCGAGATAGGCCG	qRT-PCR
XRN4-R	AGGGTGTTTTTGATCAGAC	qRT-PCR
GST-F	CACCATGTCCCCTATACTAGGTTATT	clone
GST-R	ACGCGGAACC AGATCCGATTT	clone
DCP1-F	CACCATGTCTCAAAACGGGAAGATAAT	clone
DCP1-R	TCTTTGTTGAAGTGCATTTTGTAA	clone
ACTIN2-F	AGTGGTCGTACAACCGGTATT	qRT-PCR
ACTIN2-R	GATGGCATGAGGAAGAGAGAA	qRT-PCR

Reference

1. Chang JH, Xiang S, Xiang K, Manley JL, Tong L: **Structural and biochemical studies of the 5'-->3' exoribonuclease Xrn1**. *Nature structural & molecular biology* 2011, **18**(3):270-276.
